# COVID Health Structure Index: The Vulnerability of Brazilian Microregions

**DOI:** 10.1007/s11205-021-02699-3

**Published:** 2021-05-05

**Authors:** Diogo Ferraz, Enzo Barberio Mariano, Patricia Regina Manzine, Herick Fernando Moralles, Paulo César Morceiro, Bruno Guimarães Torres, Mariana Rodrigues de Almeida, João Carlos Soares de Mello, Daisy Aparecida do Nascimento Rebelatto

**Affiliations:** 1 Department of Innovation Economics, University of Hohenheim, Wollgrasweg 23, 2nd floor, Room 520i, Stuttgart, Germany; 2 Department of Economics, Federal University of Ouro Preto (UFOP), Rua do Catete 166 Centro, Mariana/MG, 35420-000 Brazil; 3 Department of Production Engineering, São Paulo State University (UNESP), Núcleo Residencial Presidente Geisel, Avenida Engenheiro Luiz Edmundo Carrijo Coube, 14-01, Bauru, 17033360 Brazil; 4 Department of Gerontology, Federal University of São Carlos (UFSCar), Rod. Washington Luiz, s/n, São Carlos, SP 13565-905 Brazil; 5 Department of Production Engineering, Federal University of São Carlos (UFSCar), Rod. Washington Luiz, s/n, São Carlos, SP 13565-905 Brazil; 6 DST/NRF South African Chair in Industrial Development, College of Business and Economics, University of Johannesburg, 31 Henley Road, Aucklandpark, Johannesburg, 2092 South Africa; 7 Department of Production Engineering, Fluminense Federal University (UFF), Rua Passo da Pátria, Campus Praia Vermelha, Bloco D - sala 309, Niterói, 24210-240 Brazil; 8 Department of Production Engineering, Federal University of Rio Grande do Norte (UFRN), Av. Senador Salgado Filho, n° 3000, Campus Universitário Lagoa Nova - Centro de Tecnologia, Natal, 59078-970 Brazil; 9 Department of Production Engineering, University of São Paulo (EESC/USP), Av. Trab. São Carlense, 400 - Parque Arnold Schimidt, São Carlos, 13566-590 Brazil

**Keywords:** Coronavirus pandemic. health service. decision index. Brazilian microregions. Data Envelopment Analysis (DEA)

## Abstract

Many developing countries have highly unequal health systems across their regions. The pandemic of COVID-19 brought an additional challenge, as hospital structures equipped with doctors, intensive care units and respirators are not available to a sufficient extent in all regions. Using Data Envelopment Analysis, we create a COVID Index to verify whether the hospital structures in 543 Brazilian microregions are adequate to deal with COVID-19 and to verify whether public policies were implemented in the right direction. The results indicate that hospital structures in the poorest microregions were the most vulnerable, although the peak of COVID-19 occurred in the richest microregions (Sao Paulo). The Southeast states could relocate hospital resources or even patients between their regions. The relocation was not possible in many states in the Northeast, as the health system poorly assisted the interior of these states. These findings reveal that the heterogeneity of microregions’ hospital structures follows the patterns of socioeconomic inequalities. We conclude that it is easier for the wealthier regions to reallocate hospital resources internally than for the poorest regions. By using the COVID Index, policymakers and hospital managers have straightforward information to decide which regions must receive new investments and reallocate underutilized resources.

## Introduction

Developing countries tend to have inadequate hospital spending and structures to provide health services (Bein et al., 2017; Dieleman et al., 2018; Nyashanu et al., 2020). Hospital managers and public authorities face the challenge of (re)allocating the scarce equipment and human capital, especially in regions like Africa and Latin America (Araújo et al., 2014; de Almeida Botega et al., 2020; Varela et al., 2010). Moreover, countries such as Brazil have a highly unequal health structure across microregions (Coelho et al., 2020). Some regions have an adequate number of resources, while others lack essential materials to provide health services.

The coronavirus pandemic brought a new challenge to developing countries because equipment like respirators are not readily available for import. This scenario requires straightforward mechanisms to reveal which areas demand more attention from policymakers to receive investments and reallocated materials (Noronha, 2020). The study of hospital structure is crucial to provide treatment for infected people, avoid deaths, and prevent the collapse of the health network due to the coronavirus pandemic. The aim of this study was to create a new indicator, called the COVID Index, to analyze how the hospital structure has responded to the coronavirus pandemic in the Brazilian microregions.

Several studies have analyzed the efficiency of health systems and hospitals using Data Envelopment Analysis (DEA) (Blaakman et al., 2014; Hollingsworth et al., 1999; Jacobs, 2001; Kohl et al., 2019; Rouyendegh et al., 2019; Siddharthan et al., 2000; Zare et al., 2019). Hollingsworth et al. (1999) and Kohl et al. (2019) reviewed the literature to understand DEA applications in health care, with a particular focus on hospitals. Jacobs (2001) used DEA to compare the efficiency rankings of cost indices to benchmark National Health Service (NHS) hospitals in the United Kingdom. Zare et al. (2019) used a hybrid DEA and game theory to evaluate the performance and productivity of healthcare centers. The authors found that inefficient centers can improve their performance by adjusting their coverage domains and adopting flexible work hours and zone-based assignments for the specialist staff. Rouyendegh et al. (2019) used a fully-ranking DEA–FAHP approach for firms in the health care industry to investigate the business performance. The authors argue that hybrid models provide benefits considering the value of the weights determined by the data from the hybrid model. Acharya and Porwal created a vulnerability index to manage the response of the COVID-19 pandemic in India. The authors used five dimensions (socioeconomic, demographic, housing and hygiene, epidemiological, and health system) through 15 variables. They found that districts in nine large states have a high overall vulnerability (index value more than 0.75) (Acharya & Porwal, 2020).

Several authors have analyzed the Brazilian health system. De Almeida Botega et al. (2020) pointed out a high level of hospital inefficiency. Similarly, Varela et al. (2010) analyzed one of the most developed health systems in Brazil and found that only 6.41% of the municipalities in Sao Paulo state were efficient. On the other hand, Araújo et al. (2014) focused on Brazilian for-profit hospitals, which generally presented efficiency scores.

Our study differs from previous literature in three ways. First, we focus on coronavirus infections and deaths compared to the necessary hospital structure for this disease. Second, we analyze Brazilian microregions, since small municipalities depend on geographically close urban centers. Third, we advance using the metafrontier technique to analyze regions over months. Other authors previously applied the inverted DEA technique (de Barrosa & Ramosb, 2010; Silveira, 2012; Enzo Mariano et al., 2020), as well as metafrontier analysis (Chen et al., 2020).

Brazil is a good case study because the country has strong regional heterogeneity. The North and Northeast regions are less developed than the South and Southeast, where the federal units have shown more significant economic and human development indicators (Ferraz et al., 2020). Moreover, public authorities and managers have limited public funds to work with until 2027 (Constitutional Amendment 95/2016). This challenging scenario can provide findings to develop policy recommendations and guidelines for the (re)allocation of resources during the coronavirus pandemic. Previous studies have shown the differences in Brazilian regions during the pandemic. For example, the distribution of ICU beds varies from 0.07 in a macroregion of the Amazonas state to 3.3 in a macroregion in the Sao Paulo state. The macroregions with the lowest supply are mostly in the North and Northeast of Brazil. In contrast, the 30 macroregions with the greatest supply are in Sao Paulo, Parana, Santa Catarina, Rio Grande do Sul, Mato Grosso do Sul, Mato Grosso and Goiás states (Noronha et al., 2020). Coelho et al. (2020) used probabilistic models to show that, despite the Sao Paulo and Rio de Janeiro were the initial hotspots of COVID-19 spread, the North and Northeast regions present a high risk of vulnerability during the COVID-19 pandemic (Coelho et al., 2020).

The COVID Index is also useful for developing regions, since low- and middle-income countries (LMICs) are especially susceptible to their fragile health systems being overloaded as the southern hemisphere moves from summer to autumn (Coetzee & Kagee, 2020; Mendelson, 2020). This risk scenario for health systems, especially in the most vulnerable regions of LMICs, requires tools to monitor and forecast a possible collapse in regional health networks, which would give competent authorities the opportunity of taking previously mitigation of the burden on health systems based on previous experiences of countries that are coming out of the peak of the contagion curve. Such actions would include the construction of field hospitals, hardening of the lockdown – along with financial measures to support the health system and protect businesses, coordination between the national and regional governments that would ensure new powers over health services, transport and internal affairs; it would also include giving members of the armed forces powers of law enforcement (Legido-Quigley et al., 2020). Moreover, the COVID Index is appropriate for evaluating hospital structure during a second coronavirus wave, other pandemic situations, or even after the pandemic, since the indicator reveals which regions should receive new investments and how to reallocate underutilized health resources.

## Coronavirus pandemic

The coronavirus was first reported in December 2019, in Wuhan, Hubei Province, China. The zoonotic β-coronavirus disease (COVID-19) (Rothan & Byrareddy, 2020) is caused by the severe acute respiratory syndrome coronavirus 2 (SARS-CoV-2), which is a β-type coronavirus (β-CoV) that originated via natural selection (Andersen et al., 2020). This virus has spread globally and, until September 13, had recorded about 28 million cases and > 911,877 deaths in 214 countries (COVID & Team, 2020; Organization, 2020).

SARS-CoV-2 is the third major human β-CoV epidemic recorded in the twenty-first century, following the other two β-CoVs, namely SARS-CoV and Middle East respiratory syndrome coronavirus (MERS-CoV), which were identified in 2002 and 2012, respectively (Guo et al., 2020).

From the time of exposure, the median incubation period for COVID-19 is four to five days, up to 14 days (Guan et al., 2020; Lauer et al., 2020; Li et al., 2020b). The symptoms of the disease range widely and may include asymptomatic frameworks, fever, cough, fatigue, diarrhea, dyspnea, headache, hemoptysis, lymphopenia, and sputum production (Carlos et al., 2020; Huang et al., 2020). Under severe pneumonia, chest CT may present RNAaemia, acute respiratory distress syndrome (ARDS), cardiac injury, and pulmonary grand-glass opacities (Huang et al., 2020), which lead to an increase in local and systemic immune response, deepening the inflammation condition (Rothan & Byrareddy, 2020).

SARS-CoV-2 uses the angiotensin-converting enzyme 2 (ACE2) protein, like SARS-CoV, as an input receptor on human cells (Zhou et al., 2020). ACE2 is widely found on lung alveolar epithelial cells and enterocytes, which may help understand the infection routes and clinical disease characteristics (Hamming et al., 2004).

Person-to-person transmission is a likely way for spreading COVID-19, either by direct contact or through droplets spread during coughing or sneezing by a sick individual. There is no evidence of vertical transmission during pregnancy (Chen et al., 2020). Regarding breastfeeding, it is recommended to avoid direct contact as the proximity in the breast could increase the risk of SARS-CoV-2 transmission via aerosol (Favre et al., 2020). In addition, since some studies have identified SARS-CoV-2 in fecal swabs and blood (Zhang et al., 2020), it is important to test fecal and urine samples so as to develop new disease-control strategies (Rothan & Byrareddy, 2020).

The dissemination and transmission periods are not yet well defined. However, viral genetic material can be detected in the upper airways of asymptomatic or pre-symptomatic patients before the onset of symptoms (Pan et al., 2020). Studies have already shown that asymptomatic patients may spread SARS-CoV-2, and therefore this group of individuals can explain the rapid spread of the virus and also represent an essential aspect in infection control (Bai et al., 2020; Rothe et al., 2020; Yu et al., 2020). Moreover, Dogan et al. (2020) investigated the correlation between meteorological parameters and COVID-19 pandemic in New Jersey, United States. The authors found that a strong positive association of lagged humidity, air quality, PM 2.5, and previous infections with daily new cases (Doğan et al., 2020). This is an important finding because it could be used as a critical input to mitigate the rapid spread of COVID-19 across the United States and other humidity areas, such as in Amazonia/Brazil.

Although COVID-19 has a lower death rate (3.8%) than SARS-CoV (10%) and MERS-CoV (37.1%), the number of cases of the disease is ten times higher (Ahn et al., 2020; Organization, 2003, 2019, 2020).

All age groups can be affected by both the infection and the severe form of COVID-19. However, people aged ≥ 65 years who are living in care institutions are more susceptible (Heymann & Shindo, 2020). Other risk factors include hypertension, cardiovascular disease, diabetes mellitus, chronic respiratory disease, cancer, renal disease and obesity (Cai et al., 2020; Garg, 2020; Guan et al., 2020; Wu & McGoogan, 2020). Since the prevalence of comorbidities (DuGoff et al., 2014) and the risks of worsening from COVID-19 are highly age-dependent variables (Promislow, 2020), the association of these conditions can lead to higher rates of hospitalization and mortality (Verity et al., 2020), which means that this potentially fatal disease is also an important global public health concern.

The situation presented here culminates in a high demand for health services, such as hospitalization, intensive care units (ICUs), equipment, supplies and, consequently, health professionals. COVID-19 may be especially dangerous for the integrity of health systems. This is because the aforementioned high infection rate (Ahn et al., 2020; Organization, 2003, 2019, 2020) puts health workers and caregivers at high risk of infection, while healthcare-associated amplification of transmission can increase pressure on health systems (Heymann & Shindo, 2020).

Thus, support for healthcare networks should be prioritized to reduce the potential of regional health systems to collapse (Mendelson, 2020; Medicine, 2020; Li et al., 2020a). Public policies therefore are required, largely due to the lack of structure in the most impoverished areas. While rich people can maintain social distance, live in places with sanitation and use a better health system, poor regions lack access to basic needs such as piped water and sewage removal. Moreover, poor people in developing countries usually live in regions with a high level of agglomeration, such as slums (*favelas*), they use crowded public transport to get to work, and they do not have access to an adequate health structure. For this reason, poor people are more vulnerable to be infected by the coronavirus and do not receive health assistance, and this requires the attention of the authorities (Coetzee & Kagee, 2020).

## Method

### Data

We considered seven variables of the official databases from April 16 to May 8, 2020, to evaluate Brazil’s hospital structure. The National Registry of Health Facilities (CNES) provides information on health facilities, such as infrastructure and human resources (Ministério da Saúde, 2015). The Brasil.io report (2020) provides the number of infected people and deaths due to the coronavirus pandemic, using information from each state health department in Brazil. These databases capture information from 2,748 municipalities and represent 86.92% of all inhabitants of Brazil in 2020. The municipalities are divided into 543 microregions. According to Ferraz et al. (2020), a microregion is an area within a federal state that presents a form of geographic space organization defined by the following dimensions: the social process, the natural environment and the communication network. These three dimensions enable space-delimited microregions to have a regional identity (Ferraz et al., 2020). Analysis by microregions leads to a greater level of depth of understanding of the problems in a region.

We analyzed the hospital structure during the pandemic using five variables as DEA inputs from private and public hospitals. The hospital structure includes healthcare equipment (respirators, intensive care units (ICUs) and hospital beds) and human capital (physicians and nurses). We consider physicians such as clinicians, infectious disease doctors and pulmonologists. We do not consider pediatric ICUs and beds for pregnant women or for the treatment of burns, since they cannot be used to treat infected people (Almeida et al., 2020). The outputs represent two variables – the number of people infected and the number of deaths both caused by the coronavirus. According to the previous study of Mariano et al. (2020), we choose these variables, which analyzed the coronavirus pandemic in Brazil using a two-stage DEA model. These variables are summarized in Table 1.

**Table 1:** Database

Variable	Classification
Respirators	Input
Intensive care units (ICU)	Input
Hospital beds	Input
Physicians	Input
Nurses	Input
People infected by the coronavirus	Output
Coronavirus deaths	Output

### Mathematical technique

DEA is a mathematical method based on linear programming and was developed by Charnes et al. (1978). This method measures the efficiency of decision-making units (DMUs) with an empirical linear frontier. DEA reveals the maximum number of outputs that can be produced per unit of inputs. Thus, it represents the production limit determined by the input restriction (Charnes et al., 1978; Cook & Seiford, 2009; E. B. Mariano & Rebelatto, 2014).

We use inverted DEA to understand the use of hospital structures in relation to the coronavirus pandemic (Enzo Mariano et al., 2020). Therefore, the higher the COVID Index, the worse the performance. In other words, top-ranked regions are those with the worst performance. This type of analysis also demonstrates the possible collapse of the hospital infrastructure, considering the high number of coronavirus cases and deaths. This approach was used by Mariano et al. (2020).

DEA models differ mainly according to the type of returns to scale and orientation. In this study, we use the variable returns to scale (VRS) model, which identifies variation among inputs and outputs and proposing three frontier areas: (a) increasing, where outputs grow proportionately more than inputs; (b) constant, where there is proportionality between inputs and outputs; and (c) decreasing, where outputs grow proportionally less than inputs (Banker et al., 1988). VRS models have the advantage of allowing relative comparisons among regions with different hospital structures, as shown in Table 2.

**Table 2:** DEA VRS model Source Mariano and Rebelatto (2014)

Input oriented	Output oriented
$MAX\mathop \sum \limits_{i = 1}^{m} u_{i} .y_{i0} + wT$	$MIN\mathop \sum \limits_{i = 1}^{n} v_{j} .x_{j0} - w$
Subject to:	Subject to:
$\mathop \sum \limits_{j = 1}^{n} v_{j} .x_{j0} = 1$	$\mathop \sum \limits_{j = 1}^{m} u_{i} .y_{i0} = 1$
$\mathop \sum \limits_{i = 1}^{m} u_{i} .y_{ik} - \mathop \sum \limits_{j = 1}^{n} v_{j} .x_{jk} + w \le 0 for k = 1,2, \ldots ,h$	$\mathop \sum \limits_{i = 1}^{m} u_{i} .y_{ik} - \mathop \sum \limits_{j = 1}^{n} v_{j} .x_{jk} + w \le 0 for k = 1,2, \ldots ,h$
*w without sign restriction*	*w without sign restriction*

where ${\text{x}}_{{{\text{jk}}}}$ represents the level of hospital structure j in a region k; y_ik_ represents the number of coronavirus cases and deaths i in a region k; x_j0_ represents the level of hospital structure j in the region; y_i0_ represents the number of coronavirus cases and deaths i in the region; v_j_ represents the weight of the hospital structure j for the region; u_i_ represents the weight of coronavirus cases and deaths i for the region; m is the quantity of analyzed output dimensions; n is the quantity of hospital structure analyzed; and w represents the scale factor.

Using this approach, we undertook two analyses. First, we investigated Brazil’s performance showing which regions had the worst performances during the coronavirus pandemic. The country analysis is essential for the Federal Government to distribute resources across regions in Brazil. Second, we applied the DEA model for an intra-state analysis. We evaluated the performance of each state in Brazil. For example, the Sao Paulo microregion is compared with other regions in the same state. This analysis has been done previously (Despotis, 2005) and it is essential to help governors, local authorities and hospital managers reallocate resources on the basis of geographical proximity and state budgets. Third, we used the metafrontier analysis (Yang et al., 2020). Since DEA is a non-parametric method, it is not possible to compare the results of the analysis carried out in different months, and we therefore suggest using metafrontier analysis. This approach was created to compare different periods under analysis (days, months or years). This combined analysis is essential to investigate the health system's vulnerability in a certain period and the evolution during some pandemic months. In our study, each DMU corresponds to a microregion in a specific month. To analyze the microregions over time, the DEA method is applied to all microregions at the same time, regardless of the month analyzed. Due to the short period of analysis, no substantial technological changes were needed and we therefore could use the metafrontier approach. This approach contributes to the COVID Index analysis because managers and policymakers can verify whether the hospital structure has been damaged during the pandemic. We therefore analyze the situation of all microregions over time (in April and May 2020) using the metafrontier technique. This analysis is critical to understand whether regions worsened their performance over time.

### Interpretation of the COVID Index

The COVID Index is a relative indicator, varying from zero to one. The closer to zero, the better the region’s performance in using hospital infrastructure to tackle the coronavirus pandemic. The closer to 1, the worse the microregion’s performance in using hospital infrastructure to face the coronavirus. The COVID Index indicates four possible performance situations, as shown in Table 3.

**Table 3:** COVID Index situation

Situation	Score
Adequate	0.00 ≥ 0.24
Satisfactory	0.25 ≥ 0.50
Attention	0.51 ≥ 0.74
Inadequate	0.75 ≥ 1.00

The four types of intervals in Table 3 categorize the level of health infrastructure in each microregion. Regions with an adequate situation offer an appropriate number of resources to treat patients infected by coronavirus. These regions might be able to assist other regions without affecting their hospital infrastructure. For example, a region classified as adequate could receive patients from or provide human resources and respirators to other regions. Satisfactory regions have suitable resources to deal with the coronavirus pandemic. Regions requiring attention need to be observed by the authorities to avoid more coronavirus cases and deaths and cannot reduce their hospital infrastructure. Regions that are inadequate present the worst situation among the analyzed regions. For this reason, they must receive resources from new investments or reallocated equipment, and human resources from regions in an adequate situation. However, it is noteworthy that the COVID Index demonstrates a relative situation by taking into account the worst situation among the analyzed regions.

The COVID Index therefore makes the following contributions: (a) it analyzes the use of infrastructure and human capital to face the coronavirus pandemic in an aggregated way; (b) it presents a straightforward interpretation and visualization of information and analysis of temporal evolution; and (c) it provides subsidies for policymakers to (re)allocate financial resources, infrastructure and human capital more optimally during the pandemic; (d) it points out situations that need attention where confirmed cases of coronavirus and deaths increase; and (e) it anticipates the possibility of the collapse of the hospital system (attention situation), which is crucial for developing preventive policy recommendations.

## Results

Analyzing the 543 microregions in Brazil, our findings reveal that, in April, 20 microregions (3.68%) presented the worst COVID Index (1.0). Although the Southeast region and Sao Paulo state were considered the epicenter of the pandemic, 60% of the regions classified as experiencing an inadequate situation were concentrated in Brazil’s North or Northeast. This result is explained by the inadequate hospital infrastructure of these poor regions, where the health system struggles with a lack of resources and professionals. In May, 26 microregions (4.78%) presented the worst performance in terms of the COVID Index. These microregions were concentrated in the North (46.15%) and Northeast (39.62%), and this finding corroborates with previous studies that analyze the Brazilian health system during the pandemic (Coelho et al., 2020; Noronha et al., 2020). In contrast, 60% of the regions with the 20 best COVID Index performance scores (0.0 to 0.25) were concentrated in the South, Southeast and Midwest of Brazil. These findings reveal the inequality of hospital infrastructure across the Brazilian microregions, which follows the patterns of economic and social discrepancies in Brazil. The pandemic worsened the condition of the hospital network in the North and Northeast, while the South and Southeast of Brazil better managed the treatment of infected people and could reduce deaths caused by the coronavirus.

**Fig. 1:**
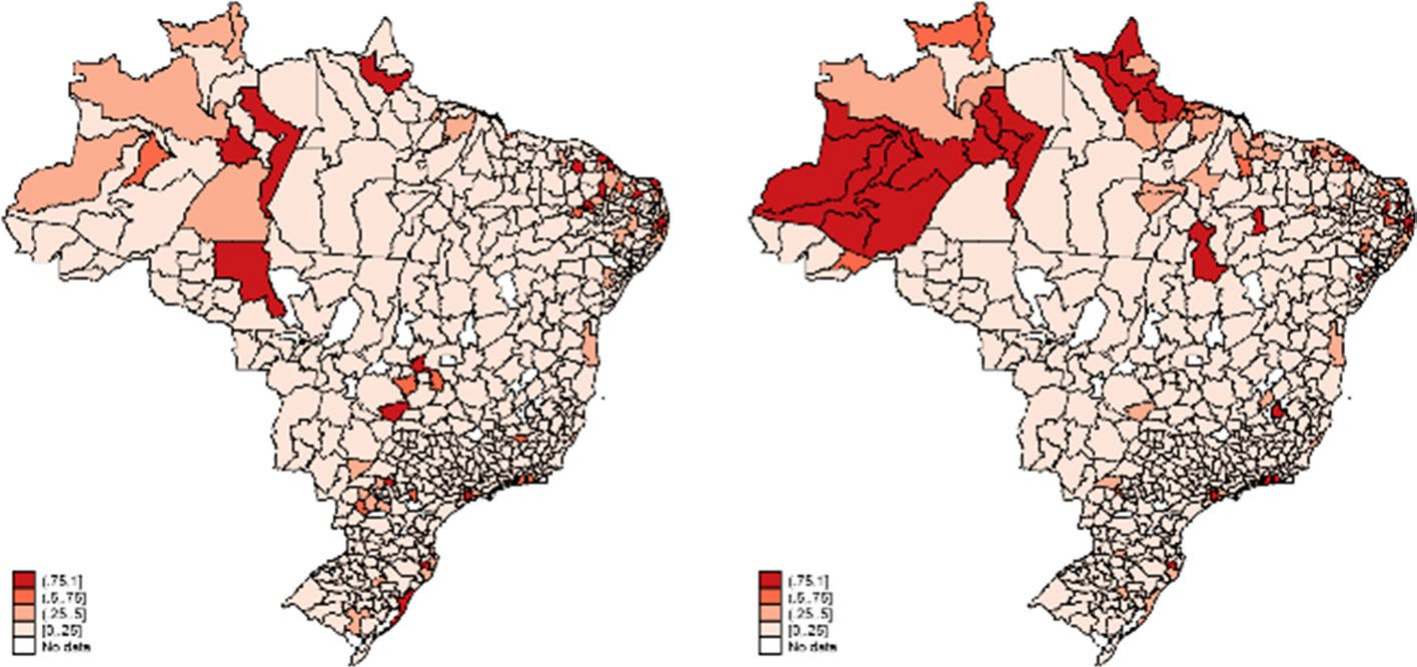
COVID Index for microregions in Brazil

Figure 1 shows the relative performance of the Brazilian microregions in the two months analyzed in this work. Dark red represents the regions with indicators close to one (inadequate situation), while light red represents the regions with indicators close to zero (adequate situation). The first map shows the distribution of the COVID Index in April. The second map shows the COVID Index in May.

Brazil has a vast territory, however, which makes it challenging to reallocate resources across regions, especially from better to worse hospital infrastructure. Moreover, the Federal Government did not institute coordination among the Brazilian states, which required interventions from the local authorities. We therefore argue that the COVID Index must also provide information comparing microregions within states. This information is useful for hospital managers to (re)distribute equipment and human resources among nearby regions.

**Fig. 2:**
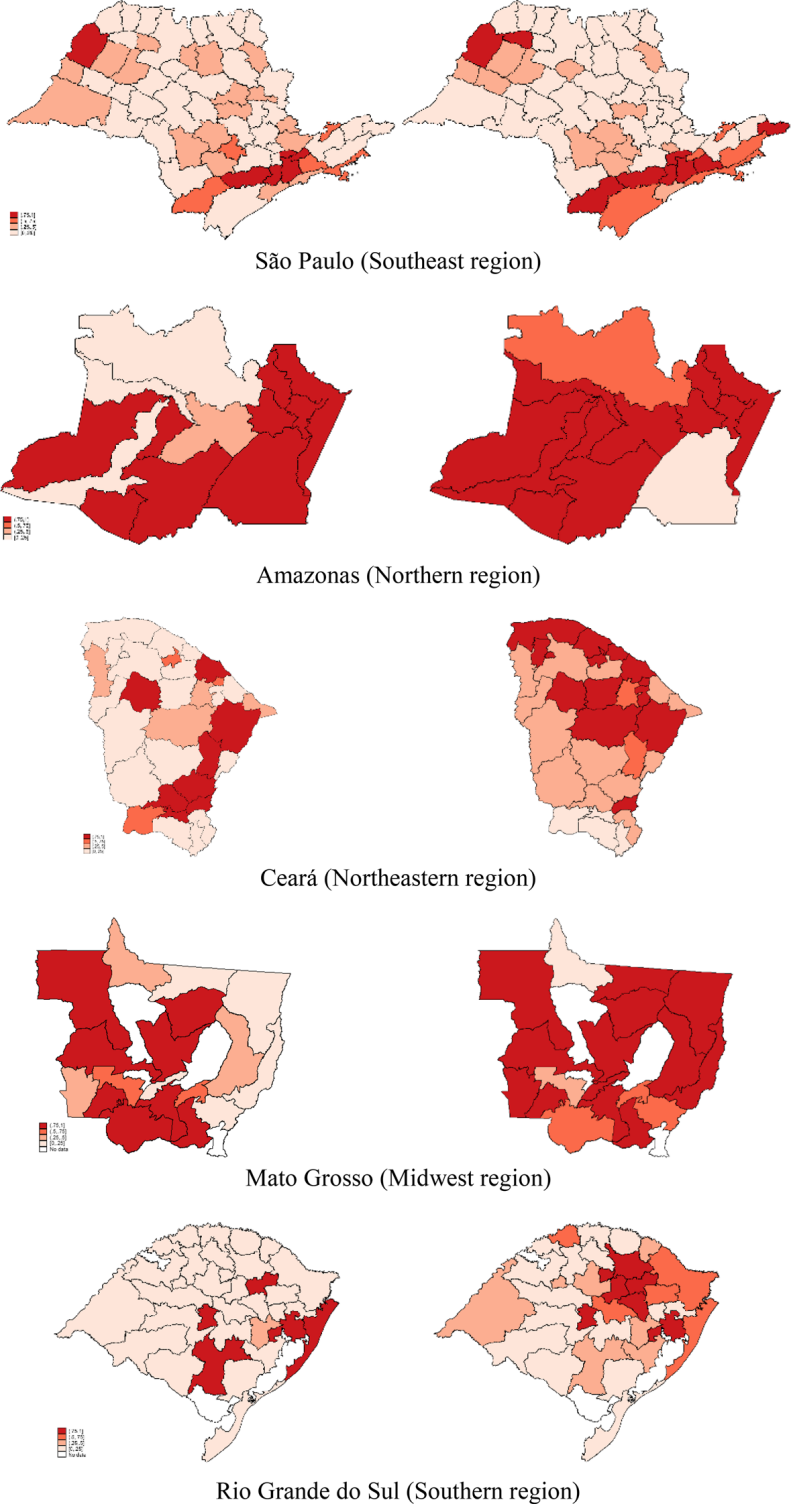
COVID Index microregions by regional evaluation

The within-state COVID Index Fig. 2 reveals some patterns. In April (on the left), the worst performance was concentrated in urban centers, especially in capitals like Sao Paulo, Rio de Janeiro, Manaus and Belém. However, in May (on the right), the worst COVID Index performance spread from urban centers to the countryside (Dom Phillips, 2020).

Moreover, several states present an increasing number of microregions in a situation that needs attention or is inadequate. For example, in April, Piauí state had only five microregions with a COVID Index equal to one. In contrast, Piauí had eleven microregions (+ 6) with a COVID Index equal to one in May. The same is found in other states, such as Alagoas (+ 5), Goiás (+ 5), Mato Grosso (+ 5) and Sergipe (+ 5). Note that these comparisons should be made only within states.

Comparing the data in greater detail, we can observe the weaknesses or otherwise of the Brazilian capitals regarding the number of respirators, physicians and beds in relation to the number of people infected. This explains why most state capitals achieved the worst performance.

The COVID Index reveals a perverse dynamic of how hospital managers and public authorities reallocate resources within the state. On the one hand, the inadequate situation in the Sao Paulo microregions (1.00) could use resources from other developed microregions in the countryside of the state, such as Campinas (0.1947), Piracicaba (0.2412) and São Carlos (0.3121). The reallocation would be possible because these regions are in an adequate or satisfactory situation. On the other hand, the Amazonas state concentrates equipment and human resources in the collapsing capital, called Manaus (1.00) (Tom Phillips & Maisonnave, 2020). However, Manaus cannot reallocate resources because the other eleven microregions in Amazonas also present an inadequate index. In Amazonas, only two microregions were in an adequate or satisfactory situation, namely Madeira (0.2245) and Rio Negro (0.3988), but geographical distance and the reduced number of resources were insufficient to provide the necessary resources to Manaus.

**Fig. 3:**
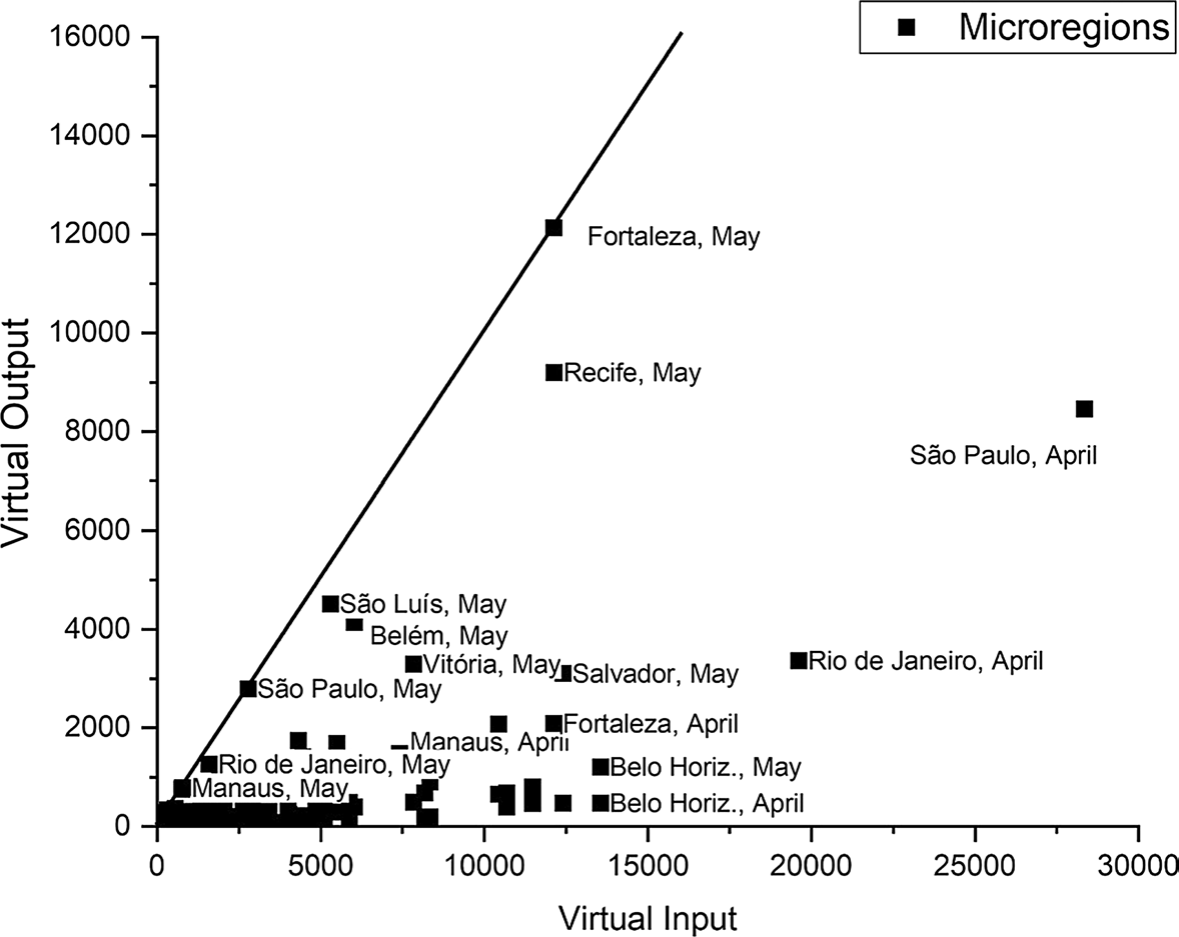
Metafrontier analysis

We used the metafrontier technique to corroborate the country and intrastate analyses, comparing the situation between April and May. In April, only three microregions presented the worst performance (1.0), viz. Anapolis (GO), Pedro Osorio (RS) and Tabuleiro (SC). In contrast, 26 microregions presented a COVID Index equal to one in May. However, the collapsing regions in May were in the North and Northeast (73.08%), which reveals that the pandemic strongly affected that region over time. The state of Amazonas was the most affected, since ten microregions (76.92%) presented an inadequate situation due to coronavirus pandemic, as shown in Fig. 3.

Table 4 shows the difference between the COVID Index in April and May, using the state average. A higher difference means worsening regional performance over time. Amazonas state presents the worst difference (0.8424), which means that the COVID Index increased (0.8424) from April (0.0425) to May (0.8849). This pattern is followed in the other 24 states and the federal district. Almost all of Brazil’s federal units worsened in the COVID Index from April to May. Goiás was the only state that improved its COVID Index performance over time.

**Table 4:** Metafrontier ranking by federal units

Federal units	Diff	Rank	Signal
Amazonas	0.8424	1	+
Amapá	0.6906	2	+
Roraima	0.3487	3	+
Pará	0.2913	4	+
Alagoas	0.2593	5	+
Ceará	0.2555	6	+
Pernambuco	0.2493	7	+
Paraíba	0.2260	8	+
Maranhão	0.2090	9	+
Sergipe	0.2062	10	+
Tocantins	0.1825	11	+
Piauí	0.1798	12	+
Acre	0.1568	13	+
Distrito Federal	0.1366	14	+
Santa Catarina	0.1325	15	+
Espírito Santo	0.1273	16	+
Rio Grande do Norte	0.1236	17	+
Rio de Janeiro	0.1072	18	+
Rondônia	0.0963	19	+
São Paulo	0.0908	20	+
Minas Gerais	0.0523	21	+
Paraná	0.0512	22	+
Bahia	0.0393	23	+
Mato Grosso	0.0311	24	+
Rio Grande do Sul	0.0274	25	+
Mato Grosso do Sul	0.0256	26	+
Goiás	− 0.0198	27	–
Brazil	0.1442	–	+

## Policy recommendations

The coronavirus pandemic gave rise to a scenario that challenged the Brazilian hospital infrastructure (Rezende Machado de Sousa et al., 2019). Policymakers, public authorities and hospital managers had to deal with the shortage of international hospital products and an undervalued exchange rate for respirator imports. This unprecedented scenario affected both private and public hospitals in Brazil. The contractionary fiscal policy also worsened the public health system, which is responsible for serving most of the Brazilian population, especially the low-income population. Surprisingly, the Federal Government and the Ministry of Health did not present a coordinated program to provide materials or hire health professionals, especially for regions close to hospital saturation (Ortega & Orsini, 2020). Regarding the lack of coordination, the Federal Government pushed the responsibility to local authorities, who face several problems, such as a limited budget and difficulty acquiring international equipment.

The Federal Government’s actions were limited to distributing financial resources, respirators and other materials to hospitals in several regions, especially in urban centers. This distribution seems subjective or questionable, since some rich states received more attention than poor regions with a lack of hospital infrastructure. For example, the Federal Program to Combat Coronavirus (*Programa Federativo de Enfrentamento ao Coronavírus*) created by the Federal Government allocated the fixed amount of 60 billion Reais (R$) [U$11,353 billion] from the Federal Government to states and municipalities. According to the law, authorities can spend R$ 50 billion [U$ 9,461 billion] on several economic purposes. Authorities are obligated to spend R$ 10 billion [U$ 1,892 billion] on health assistance. However, only R$ 2.8 billion [U$ 529 million] were destinated taking into account the number of people infected by coronavirus. In addition, the Federal Government transferred R$ 92 million [U$17,408 million] to Sao Paulo state. This financial resource was available to increase the number of hospital beds. However, the COVID Index shows that the Sao Paulo countryside had resources available, since 46.03% of microregions were classified as being in a comfortable situation. The Federal Government could provide resources to regions in the North and Northeast, where the hospital infrastructure was relatively speaking worse than in Brazil’s wealthy areas. For example. the federal authority should have transferred financial resources to Amazonas, where hospitals in 84.61% of the microregions were classified as inadequate. Moreover, the Ministry of Health distributed 20 respirators to Pará state, where the pandemic collapsed the primary urban centre’s hospital infrastructure and the countryside is not in a situation to support the capital. As part of the same program, the Ministry of Health also distributed 20 respirators to Paraná state, the developed region in the South, where 25.71% of the microregions were classified as being in an adequate situation in May. In this sense, we argue that the Federal Government should use straightforward mechanisms to distribute or reallocate health resources across the country during the pandemic. If performance intervals were used, the minister could make decisions based on the entire Brazilian health infrastructure.

In addition, the state authorities in developed regions did not present plans to reallocate equipment and human resources. For example, when the hospital infrastructure in Sao Paulo city was under pressure, the authorities could have obtained physicians and nurses from its countryside, considering that the coronavirus had not yet spread. Besides, the occupation of beds and ICUs in the Sao Paulo countryside hospitals was much lower than in the capital. It is noteworthy that 19.5% of the microregions in Sao Paulo state were in an adequate or satisfactory situation in May, followed by the states of Minas Gerais (17.43%), Rio Grande do Sul (8.71%), Bahia (8.71%) and Paraná (7.47%). Policymakers therefore need adequate regional information to reallocate resources and to give instructions to transfer patients between hospitals when they are close to collapse.

**Fig. 4:**
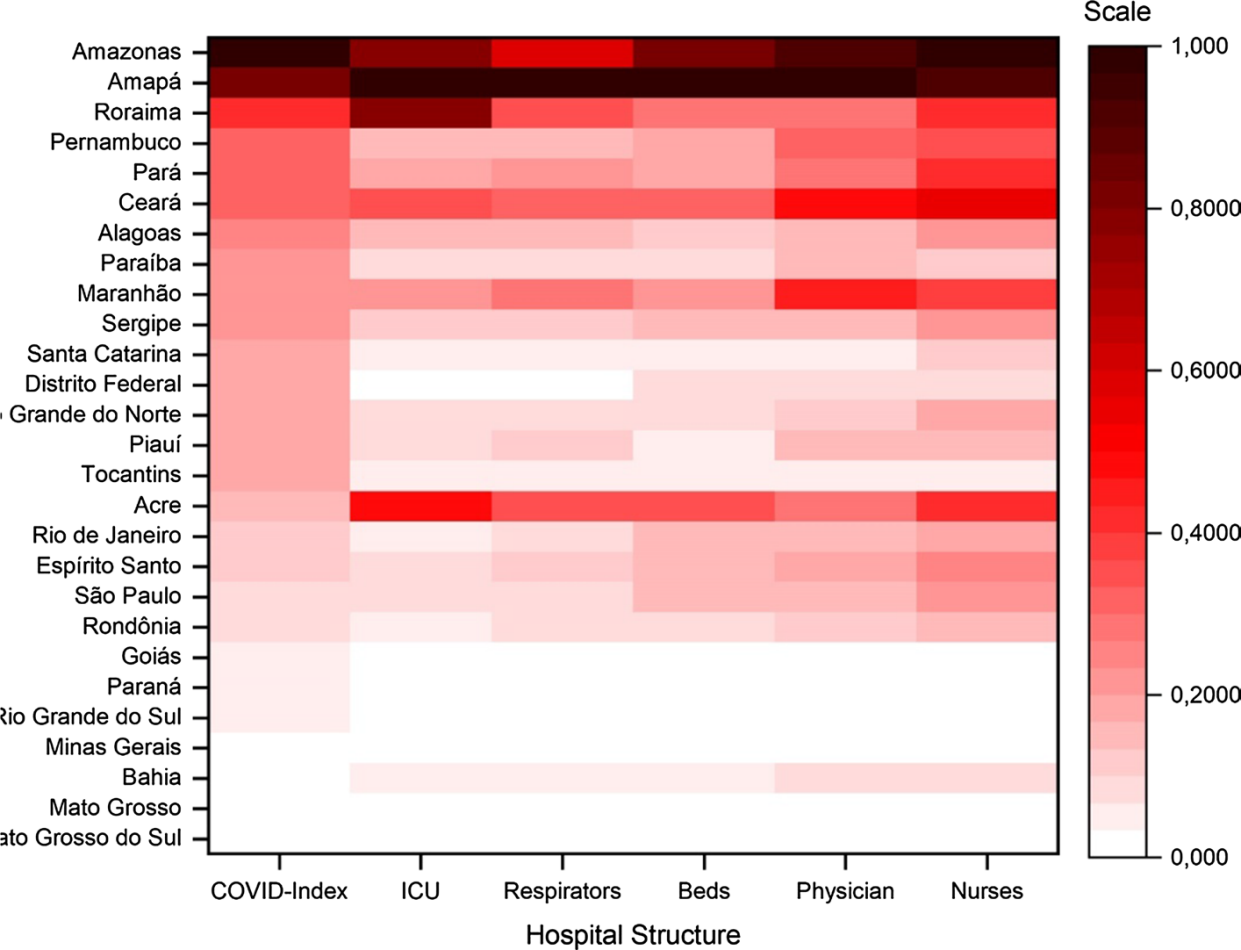
Hospital infrastructure for number of infected people

Furthermore, several policymakers and managers concentrated their efforts on importing respirators. Figure 4 shows that collapsing regions also experienced a high demand for ICUs, beds, physicians and nurses because of the number of infected people. Due to the scarcity of several resources, the local authorities faced a complex environment in which to cope with the pandemic. Managers need to improve the number of various hospital resources.

**Fig. 5:**
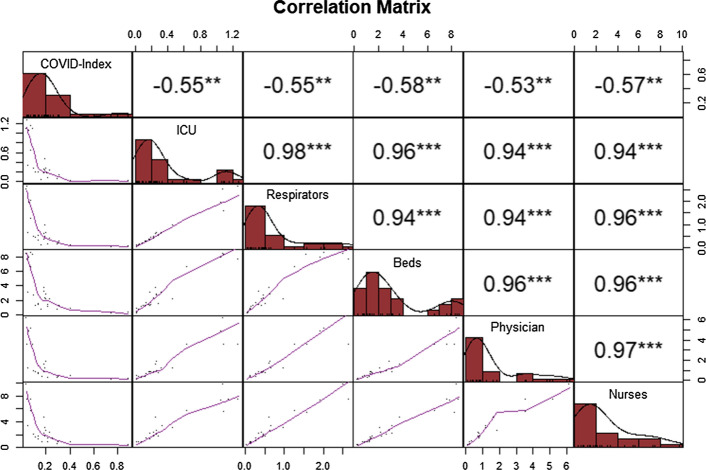
Correlation matrix

Comparing the correlation between the COVID Index and the demand for hospital infrastructure by number of infected people, Fig. 5 shows that all resources are negatively correlated with our indicator. However, the correlation matrix shows that these resources present a similar correlation. For example, the negative correlation between the use of ICU and the COVID Index was − 55%, which is close to the correlations of respirators (− 55%), beds (− 58%), physicians (− 53%) and nurses (− 57%).

Finally, public and private hospital managers failed to promote new collaborations during the pandemic. For example, several public hospitals in urban centers were overcrowded, while private hospitals had beds and equipment available (Barbara, 2020). Public–private partnerships could solve this issue, especially in urban centers like Sao Paulo, Manaus and Belém, and a few public authorities have resorted to this type of alternative.

## Concluding remarks

We created a COVID Index to analyze the hospital infrastructure in 543 Brazilian microregions during the coronavirus pandemic. The COVID Index reveals that microregions in the North and Northeast of Brazil need more attention from policymakers. Also, the metafrontier shows that the Brazilian situation became worse from April to May 2020. However, as is the case in Sao Paulo state, rich regions have better hospital infrastructure than in the countryside, which provides an alternative for reallocating resources.

Some limitations of the study, presented here, could open multiple avenues for future research. First, there are underreported cases of infected people in Brazil, which occurs due to the small number of people tested. This makes it clear that the government needs to increase the number of tests it does. Second, this study does not consider the number of beds offered by the field hospitals created during the pandemic. This data is not available for microregions on Datasus. The Ministry of Health should disseminate this information more quickly so that scientists can carry out studies and propose measures that minimize the impacts of the new coronavirus. Third, we do not discuss the quality of health resources, which can affect the number of deaths. Moreover, future studies might incorporate period effects using dynamic models, along with the DEA-GSZ model, Malmquist index, the DEA–FAHP approach, DEA with two-stage models, and indexes taking into account a great number of variables (socioeconomic, infrastructure, income distribution, among others). These DEA models and variables can corroborate the hospital network’s optimization during the pandemic (Kanematsu et al., 2020; Lins et al., 2003; Mariz et al., 2018).

Finally, this study contributes to policymakers’ and hospital managers’ understanding of which regions are most affected by the pandemic. Through the COVID Index, policymakers have straightforward information to decide which regions must receive new investments, hire health professionals, and reallocate resources. Moreover, this indicator collaborates with hospital managers to understand where there are potential underutilized resources, which is essential for resource reallocation. We argue that the COVID Index is a useful tool for countries with unequal regions, especially developing countries, and that it could be used even after the pandemic. Policymakers and hospital managers can use this indicator to assist in regional and national hospital planning to optimize patients’ treatment.

## Data Availability

Data and material are from public databases. They are available upon request. Code is not available

## References

[CR1] Acharya R, Porwal A (2020). A vulnerability index for the management of and response to the COVID-19 epidemic in India: an ecological study. The Lancet Global Health.

[CR2] Ahn, D.-G., Shin, H.-J., Kim, M.-H., Lee, S., Kim, H.-S., Myoung, J., et al. (2020). Current status of epidemiology, diagnosis, therapeutics, and vaccines for novel coronavirus disease 2019 (COVID-19). 10.4014/jmb.2003.03011. 10.4014/jmb.2003.03011 PMC9728410 32238757

[CR3] Almeida, J., Conceição, S., Pinto, L., Magalhães, V., Nascimento, I., & Costa, M. (2020). Previsão de disponibilidade de leitos nos estados brasileiros e Distrito Federal em função da pandemia de COVID-19, situação de leitos SUS e não-SUS. Belo Horizonte: Laboratório de Tecnologia de Apoio à Decisão em Saúde, NESCOM, UFMG; [cited 2020 May 7].(Nota Técnica; nº 7).

[CR4] Andersen KG, Rambaut A, Lipkin WI, Holmes EC, Garry RF (2020). The proximal origin of SARS-CoV-2. Nature medicine.

[CR5] Araújo C, Barros CP, Wanke P (2014). Efficiency determinants and capacity issues in Brazilian for-profit hospitals. Health care management science.

[CR6] Bai Y, Yao L, Wei T, Tian F, Jin D-Y, Chen L (2020). Presumed asymptomatic carrier transmission of COVID-19. JAMA.

[CR7] Banker, R. D., Charnes, A., Cooper, W. W., & Maindiratta, A. (1988). *A comparison of DEA and translog estimates of production frontiers using simulated observations from a known technology. Applications of modern production theory: Efficiency and productivity* (pp. 33–55). Springer. 10.1007/978-94-009-3253-1_2.

[CR8] Bein MA, Unlucan D, Olowu G, Kalifa W (2017). Healthcare spending and health outcomes: Evidence from selected East African countries. African health sciences.

[CR9] Blaakman AP, Salehi AS, Boitard R (2014). A cost and technical efficiency analysis of two alternative models for implementing the basic package of health services in Afghanistan. Global public health.

[CR10] Cai Q, Chen F, Wang T, Luo F, Liu X, Wu Q (2020). Obesity and COVID-19 severity in a designated hospital in Shenzhen. China. Diabetes care.

[CR11] Carlos WG, Dela Cruz CS, Cao B, Pasnick S, Jamil S (2020). Novel Wuhan (2019-nCoV) Coronavirus. American Journal of Respiratory and Critical Care Medicine Respir Crit Care Med.

[CR12] Charnes A, Cooper WW, Rhodes E (1978). Measuring efficiency of decision-making units. European Journal of Operational Research.

[CR13] Chen H, Guo J, Wang C, Luo F, Yu X, Zhang W (2020). Clinical characteristics and intrauterine vertical transmission potential of COVID-19 infection in nine pregnant women: A retrospective review of medical records. The Lancet.

[CR14] Coelho FC, Lana RM, Cruz OG, Villela DA, Bastos LS, Pastore y, Piontti A, (2020). Assessing the spread of COVID-19 in Brazil: Mobility, morbidity and social vulnerability. PLoS ONE.

[CR15] Coetzee BJ, s., & Kagee, A. (2020). Structural barriers to adhering to health behaviours in the context of the COVID-19 crisis considerations for low-and middle-income countries. Global Public Health.

[CR16] Cook WD, Seiford LM (2009). Data envelopment analysis (DEA)–Thirty years on. European journal of operational research.

[CR17] COVID, T. C., & Team, R (2020). Severe outcomes among patients with coronavirus disease 2019 (COVID-19)-United States, February 12-March 16, 2020. MMWR. Morbidity and Mortality Weekly Report.

[CR18] de Barrosa TD, Ramosb TG (2010). Avaliação dos atrasos em transporte aéreo com um modelo DEA. Production.

[CR19] de Almeida Botega L, Andrade MV, Guedes GR (2020). Brazilian hospitals’ performance: An assessment of the unified health system (SUS). Health Care Management Science.

[CR20] Despotis D (2005). A reassessment of the human development index via data envelopment analysis. Journal of the Operational Research Society.

[CR21] Dieleman JL, Sadat N, Chang AY, Fullman N, Abbafati C, Acharya P (2018). Trends in future health financing and coverage: future health spending and universal health coverage in 188 countries, 2016–40. The Lancet.

[CR22] Doğan B, Jebli MB, Shahzad K, Farooq TH, Shahzad U (2020). Investigating the effects of meteorological parameters on COVID-19: Case study of New Jersey. United States. Environmental Research.

[CR23] DuGoff EH, Canudas-Romo V, Buttorff C, Leff B, Anderson GF (2014). Multiple chronic conditions and life expectancy: A life table analysis. Medical care.

[CR24] Favre G, Pomar L, Qi X, Nielsen-Saines K, Musso D, Baud D (2020). Guidelines for pregnant women with suspected SARS-CoV-2 infection. The Lancet Infectious Diseases.

[CR25] Ferraz D, Mariano EB, Rebelatto D, Hartmann D (2020). Linking human development and the financial responsibility of regions: Combined index proposals using methods from data envelopment analysis. Social Indicators Research.

[CR26] Garg S (2020). Hospitalization rates and characteristics of patients hospitalized with laboratory-confirmed coronavirus disease 2019—COVID-NET, 14 States, March 1–30, 2020.

[CR27] Guan W-J, Ni Z-Y, Hu Y, Liang W-H, Ou C-Q, He J-X (2020). Clinical characteristics of coronavirus disease 2019 in China. New England journal of medicine.

[CR28] Guo Y-R, Cao Q-D, Hong Z-S, Tan Y-Y, Chen S-D, Jin H-J (2020). The origin, transmission and clinical therapies on coronavirus disease 2019 (COVID-19) outbreak–an update on the status. Military Medical Research.

[CR29] Hamming I, Timens W, Bulthuis M, Lely A, Navis G, v., & van Goor, H. (2004). Tissue distribution of ACE2 protein, the functional receptor for SARS coronavirus A first step in understanding SARS pathogenesis. The Journal of Pathology: A Journal of the Pathological Society of Great Britain and Ireland.

[CR30] Heymann DL, Shindo N (2020). COVID-19: what is next for public health?. The Lancet.

[CR31] Hollingsworth B, Dawson PJ, Maniadakis N (1999). Efficiency measurement of health care: A review of non-parametric methods and applications. Health care management science.

[CR32] Huang C, Wang Y, Li X, Ren L, Zhao J, Hu Y (2020). Clinical features of patients infected with 2019 novel coronavirus in Wuhan. China. The lancet.

[CR33] Jacobs R (2001). Alternative methods to examine hospital efficiency: Data envelopment analysis and stochastic frontier analysis. Health care management science.

[CR34] Kanematsu SY, Carvalho NP, Martinhon CA, Almeida MR (2020). Ranking using η-efficiency and relative size measures based on DEA. Omega.

[CR35] Kohl S, Schoenfelder J, Fügener A, Brunner JO (2019). The use of Data Envelopment Analysis (DEA) in healthcare with a focus on hospitals. Health care management science.

[CR36] Lauer SA, Grantz KH, Bi Q, Jones FK, Zheng Q, Meredith HR (2020). The incubation period of coronavirus disease 2019 (COVID-19) from publicly reported confirmed cases: estimation and application. Annals of internal medicine.

[CR37] Legido-Quigley H, Mateos-García JT, Campos VR, Gea-Sánchez M, Muntaner C, McKee M (2020). The resilience of the Spanish health system against the COVID-19 pandemic. The lancet public health.

[CR38] Li L, Xv Q, Yan J (2020). COVID-19: the need for continuous medical education and training. The Lancet Respiratory Medicine.

[CR39] Li Q, Guan X, Wu P, Wang X, Zhou L, Tong Y (2020). Early transmission dynamics in Wuhan, China, of novel coronavirus–infected pneumonia. New England Journal of Medicine.

[CR40] Lins MPE, Gomes EG, de Mello JCCS, de Mello AJRS (2003). Olympic ranking based on a zero sum gains DEA model. European Journal of Operational Research.

[CR41] Machado R, de Sousa L, Saint Ville A, Maria Segall-Corrêa A, Melgar-Quiñonez H (2019). Health inequalities and well-being in times of financial and political crisis in Brazil, a case study. Global Public Health.

[CR42] Mariano EB, Rebelatto DAD (2014). Transformation of wealth produced into quality of life: Analysis of the social efficiency of nation-states with the DEA's triple index approach (Article). Journal of the Operational Research Society.

[CR43] Mariano, E., Torres, B., de Almeida, M. R., Ferraz, D., Rebelatto, D. A., & de Mello, J. C. S. (2020). Brazilian states in the context of COVID-19 pandemic: An index proposition using Network Data Envelopment Analysis. *IEEE Latin America Transactions*, 100(1e).

[CR44] Mariz FB, Almeida MR, Aloise D (2018). A review of dynamic data envelopment analysis: State of the art and applications. International Transactions in Operational Research.

[CR45] Medicine TLR (2020). COVID-19: delay, mitigate, and communicate. The Lancet. Respiratory Medicine.

[CR46] Mendelson M (2020). Could enhanced influenza and pneumococcal vaccination programs help limit the potential damage from SARS-CoV-2 to fragile health systems of southern hemisphere countries this winter?. International Journal of Infectious Diseases.

[CR47] Noronha KVM, d. S., Guedes, G. R., Turra, C. M., Andrade, M. V., Botega, L., Nogueira, D., (2020). Pandemia por COVID-19 no Brasil: análise da demanda e da oferta de leitos hospitalares e equipamentos de ventilação assistida segundo diferentes cenários. Cadernos de Saúde Pública.

[CR48] Nyashanu M, Simbanegavi P, Gibson L (2020). Exploring the impact of COVID-19 pandemic lockdown on informal settlements in Tshwane Gauteng Province. South Africa. Global Public Health.

[CR49] Organization, W. H. (2003). Summary of probable SARS cases with onset of illness from 1 November 2002 to 31. http://www.who.int/csr/sars/country/table2004_04_21/en/index.html.

[CR50] Organization, W. H. (2019) 'Middle East respiratory syndrome coronavirus (MERS-CoV)'.

[CR51] Organization, W. H. (2020). Report of the WHO-China Joint Mission on coronavirus disease 2019 (COVID-19). *Significant account of fatality rates and comorbidities in reports from China related to COVID-19 infection*.

[CR53] Ortega F, Orsini M (2020). Governing COVID-19 without government in Brazil: Ignorance, neoliberal authoritarianism, and the collapse of public health leadership. Global public health.

[CR54] Pan Y, Zhang D, Yang P, Poon LL, Wang Q (2020). Viral load of SARS-CoV-2 in clinical samples. The Lancet Infectious Diseases.

[CR55] Phillips, D. (2020). Brazil’s coronavirus catastrophe is spreading into the country’s vulnerable interior. The Intercept https://theintercept.com/2020/07/21/coronavirusbrazil-interior-bolsonaro.

[CR56] Phillips, T., & Maisonnave, F. (2020). Utter disaster’: Manaus fills mass graves as Covid-19 hits the Amazon. The Guardian, 30.

[CR57] Promislow DE (2020). A geroscience perspective on COVID-19 mortality.

[CR58] Rothan HA, Byrareddy SN (2020). The epidemiology and pathogenesis of coronavirus disease (COVID-19) outbreak. Journal of autoimmunity.

[CR59] Rothe C, Schunk M, Sothmann P, Bretzel G, Froeschl G, Wallrauch C (2020). Transmission of 2019-nCoV infection from an asymptomatic contact in Germany. New England Journal of Medicine.

[CR60] Rouyendegh BD, Oztekin A, Ekong J, Dag A (2019). Measuring the efficiency of hospitals: A fully-ranking DEA–FAHP approach. Annals of Operations Research.

[CR61] Siddharthan K, Ahern M, Rosenman R (2000). Data envelopment analysis to determine efficiencies of health maintenance organizations. Health Care Management Science.

[CR62] Silveira JQ, d, Meza L A, & Mello J C C B S d, (2012). Identificação de benchmarks e anti-benchmarks para companhias aéreas usando modelos DEA e fronteira invertida. Production.

[CR63] Varela PS, de Andrade Martins G, Fávero LPL (2010). Production efficiency and financing of public health: An analysis of small municipalities in the state of São Paulo—Brazil. Health Care Management Science.

[CR64] Verity R, Okell LC, Dorigatti I, Winskill P, Whittaker C, Imai N (2020). Estimates of the severity of coronavirus disease 2019: a model-based analysis. The Lancet infectious diseases..

[CR65] Wu Z, McGoogan JM (2020). Characteristics of and important lessons from the coronavirus disease 2019 (COVID-19) outbreak in China: Summary of a report of 72 314 cases from the Chinese center for disease control and prevention. JAMA.

[CR66] Yang J, Cheng J, Huang S (2020). CO2 emissions performance and reduction potential in China’s manufacturing industry: A multi-hierarchy meta-frontier approach. Journal of Cleaner Production.

[CR67] Yu P, Zhu J, Zhang Z, Han Y (2020). A familial cluster of infection associated with the 2019 novel coronavirus indicating possible person-to-person transmission during the incubation period. The Journal of infectious diseases.

[CR68] Zare H, Tavana M, Mardani A, Masoudian S, Saraji MK (2019). A hybrid data envelopment analysis and game theory model for performance measurement in healthcare. Health care management science.

[CR69] Zhang W, Du R-H, Li B, Zheng X-S, Yang X-L, Hu B (2020). Molecular and serological investigation of 2019-nCoV infected patients: Implication of multiple shedding routes. Emerging microbes & infections.

[CR70] Zhou P, Yang XL, Wang XG, Hu B, Zhang L, Zhang W (2020). A pneumonia outbreak associated with a new coronavirus of probable bat origin. Nature.

